# New Coronavirus (SARS-CoV-2): advances to flatten the curve the prison population

**DOI:** 10.1590/0037-8682-0219-2020

**Published:** 2020-05-20

**Authors:** Marcos André de Matos

**Affiliations:** 1 Universidade Federal de Goiás, Programa de Pós-Graduação Stricto Sensu em Cuidado em Enfermagem, Goiânia, GO, Brasil.

The COVID-19 pandemic is challenging, in various dimensions, for government health agencies, the academia, and security and justice services worldwide^1^. Currently, Brazil has 773,151 individuals who are imprisoned, with the prison occupancy being almost three times the total capacity; in the central region of Brazil, the rate is even higher^2^. The phenomenon of overcrowding in Brazilian prisons reveals the presence of a flawed and weak penal system, with the unbridled expansion of the prison population resulting in disorders in the prison system. This leads to incalculable risks to the health of imprisoned individuals and, consequently, of their families who visit them and the workers who provide services in these environments^3^.

Prisons, due to their architectural and structural characteristics, are potential epicenters for infectious diseases, especially HIV, hepatitis B and C, syphilis, influenza, and tuberculosis, and, at this moment, a potential source of severe acute respiratory syndrome coronavirus 2 (SARS-CoV-2) infection^4^ ^-^ ^6^. Prisons are unhealthy and overcrowded, with inefficient ventilation, poor health services, and a large proportion of individuals susceptible to COVID-19, especially those most at risk of complications, such as older adults, smokers, drug users, obese prisoners, and those with noncommunicable comorbidities, especially cardiovascular and respiratory problems^7^ ^-^ ^8^. This group’s vulnerability to the coronavirus, the stigma, society’s lack of empathy, and the divergence between the attributions of the security team and health providers further aggravate the situation, resulting in the minimization of the efforts of Public Health Policies in this context^3^.

To respond to this crisis, we need to consider prisons as reservoirs that could lead to resurgence of the epidemic if it not adequately addressed. The interrelationship between prison-system health and public health is a global reality, with several countries presenting their successful experiences in facing the pandemic in the prison system^8^ ^,^ ^9^. Brazil, considering the experience of the pandemic in other countries, anticipated the problems and implemented various initiatives and measures to reduce the spread of the virus. The National Penitentiary Department^10^ immediately invested millions of reais on measures to combat the pandemic; mandated the suspension of the entry of food and visitors, with the exception of visits on judicial requests, emergency visits, and those that by their nature cannot be postponed; relaxed prison sentencing, especially for those at risk; instituted the use of videoconferences for social, legal, educational and religious contact; and created a committee to face the crisis of the new coronavirus and an institutional website with access to data in real time to strengthen the institutional information network necessary to control the pandemic^10^.

Despite these measures, which were considered effective, by May 11, 2020, 368 suspected cases had been reported in the Brazilian prison system, with 531 confirmed cases and 22 deaths resulting from COVID-19^11^. The majority of the individuals with the infection had been out of the prison unit, which highlights the need to maintain and strengthen the measures already adopted, especially exclusive social isolation, which has been found to be effective and easy to perform. However, the disarticulation, most likely due to political-party issues, between government sectors in Brazil and international bodies, makes it difficult to implement the national plan to fight the coronavirus and potentiates the economic, social and emotional crises being experienced.

It is therefore crucial that the healthcare system take rapidly action to plan for this escalation in the demand the facing the pandemic and determine how services can be reconfigured. It is believed that interventions, in line with the experiences of other countries and government agencies, will help flatten the COVID-19 epidemic curve in the prison system.
